# Navigating disruption in the PID landscape: embracing opportunities and anticipating threats in the next ten years

**DOI:** 10.3389/fimmu.2025.1596971

**Published:** 2025-06-17

**Authors:** Lúcia Mamede, Roser Cantenys Sabà, Samya Van Coillie, Johan Prévot, Silvia Sánchez-Ramón, Cecilia Poli, Anne Barasa, Björn W. Schuller, Ayal Hendel, Nicolas Garcelon, Cornelis Boersma, Pamela Lee, Claire Booth, Luigi D. Notarangelo, Jose Drabwell, Nicholas L. Rider, Frank J. T. Staal, Siobhan O. Burns, Martin Van Hagen, Martine Pergent, Jacques G. Rivière, Nizar Mahlaoui

**Affiliations:** ^1^ International Patient Organisation for Primary Immunodeficiencies (IPOPI)I, Brussels, Belgium; ^2^ Catalan Health Service, Barcelona, Spain; ^3^ Digitalization for the Sustainability of the Healthcare System (DS3) research group – Instituto de Investigación Biomédica de Bellvitge (IDIBELL), Barcelona, Spain; ^4^ Department of Clinical Immunology, Instituto de Medicina de Laboratorio (IML) and Health Research Institute of the Hospital Clínico San Carlos (IdISSC), Madrid, Spain; ^5^ Facultad de Medicina Clínica Alemana de Santiago, Universidad del Desarrollo, Santiago, Chile; ^6^ Programa de Inmunogenética e Inmunología Traslacional, Instituto de Ciencias e Innovación en Medicina, Facultad de Medicina, Santiago, Chile; ^7^ Unidad de Inmunología y Reumatología, Hospital Roberto del Río, Santiago, Chile; ^8^ Immunology Unit, Department of Human Pathology, University of Nairobi, Nairobi, Kenya; ^9^ CHI – the Chair of Health Informatics, Technical University of Munich University Hospital, Munich, Germany; ^10^ Munich Data Science Institute (MDSI), München, Germany; ^11^ the Munich Center for Machine Learning (MCML), München, Germany; ^12^ Institute of Nanotechnology and Advanced Materials, The Goodman Faculty of Life Sciences, Bar-Ilan University, Ramat Gan, Israel; ^13^ Université de Paris, Imagine Institute, Data Science Platform, INSERM UMR 1163, Paris, France; ^14^ Health-Ecore B.V., Zeist, Netherlands; ^15^ Unit of Global Health, Department of Health Sciences, University Medical Center Groningen (UMCG), University of Groningen, Groningen, Netherlands; ^16^ Department of Management Sciences, Open University, Heerlen, Netherlands; ^17^ Department of Paediatrics and Adolescent Medicine, LKS Faculty of Medicine, The University of Hong Kong, Hong Kong, Hong Kong SAR, China; ^18^ Department of Paediatric Immunology and Gene Therapy, Great Ormond Street Hospital, London, United Kingdom; ^19^ Infection, Immunity and Inflammation Department, UCL Great Ormond Street Institute of Child Health, London, United Kingdom; ^20^ Laboratory of Clinical Immunology and Microbiology, National Institute of Allergy and Infectious Diseases, National Institutes of Health, Bethesda, MD, United States; ^21^ Department of Health Systems and Implementation Science, Virginia Tech Carilion School of Medicine, Roanoke, VA, United States; ^22^ The Department of Pediatrics, Pediatric Stem Cell Transplantation Program, Willem-Alexander Children’s Hospital, Leiden, Netherlands; ^23^ The Department of Immunology, Leiden University Medical Center, Leiden, Netherlands; ^24^ Department of Immunology, Royal Free London NHS Foundation Trust, London, United Kingdom; ^25^ Institute of Immunity and Transplantation, University College London, London, United Kingdom; ^26^ Departments of Internal Medicine, Division of Allergy & Clinical Immunology, Immunology, Erasmus University Medical Center Rotterdam, Rotterdam, Netherlands; ^27^ Department of Microbiology, Chulalongkorn University, Bangkok, Thailand; ^28^ Department of Internal Medicine, Clinical Immunology, Cipto Hospital, Universitas Indonesia, Jakarta, Indonesia; ^29^ Universitat Autònoma de Barcelona (UAB), Barcelona, Catalonia, Spain; ^30^ Infection and Immunity in Pediatric Patients Research Group, Vall d’Hebron Institut de Recerca (VHIR), Barcelona, Catalonia, Spain; ^31^ Pediatric Infectious Diseases and Immunodeficiencies Unit, Hospital Infantil I de La Dona Vall d’Hebron, Vall d’Hebron Barcelona Hospital Campus, Barcelona, Catalonia, Spain; ^32^ Jeffrey Modell Diagnostic and Research Center for Primary Immunodeficiencies, Barcelona, Catalonia, Spain; ^33^ Pediatric Hematology-Immunology and Rheumatology Unit, Necker-Enfants malades University Hospital, Assistance Publique-Hôpitaux de Paris (AP-HP), Paris, France; ^34^ French National Reference Center for Primary Immune Deficiencies (CEREDIH), Necker-Enfants malades University Hospital, Assistance Publique-Hôpitaux de Paris (AP-HP), Paris, France

**Keywords:** primary immunodeficiencies (PIDs), inborn errors of immunity (IEI), immunoglobulin replacement therapy (IGRT), targeted therapies, gene therapy (GT), digital health, artificial intelligence - AI, patient-reported outcome measures (PROMs)

## Abstract

**Introduction:**

The International Patient Organisation for Primary Immunodeficiencies (IPOPI) held its third edition of the Global Multi-Stakeholders’ Summit, gathering key primary immunodeficiencies (PID) stakeholders and experts to discuss and foment global collaboration.

**Methods:**

This edition focused on the impact of genomic medicine in PID treatment, the role of digital health, including artificial intelligence, in PID care, and how to anticipate and minimise risks to ensure optimal patient access to care.

**Results:**

These discussions aimed to examine current hurdles and brainstorm feasible solutions and priorities for the PID community in these areas in the next ten years.

**Discussion:**

These discussions led to recommendations for comprehensive approaches to care and access to treatment for PID patients, suggesting actions that will bring the community closer to treatments based on real-world evidence and adjusted to patient’s needs. To accomplish this, collaboration between academia, industry, regulatory authorities, and patients is crucial.

## Introduction

Primary immunodeficiencies (PID), also referred to as Inborn Errors of Immunity (IEI) or more recently as Primary Immune Disorders, are a heterogeneous group of conditions that reflect disorders of the immune system, characterized by recurrent infections as well as atopy, autoimmunity, autoinflammatory disorders and an increased risk of malignancies (1, 2). The International Patient Organization for Primary Immunodeficiencies (IPOPI) has been working for years at a global level in collaboration with multiple stakeholders to increase awareness of these rare disorders, including advocating for improved patient quality of life (QoL), adequate clinical management, research, and s and equitable access to innovative therapies (2, 3). To this end, IPOPI hosts multiple events to discuss current hurdles and brainstorm possible solutions and priorities for the PID community (2, 3). The Global Multi-Stakeholders’ Summit is one such event with the objective to challenge key stakeholders and experts to discuss venues and priorities, thus aligning the field to join efforts (2, 3).

The third edition of the Global Multi-Stakeholders’ Summit was held in 2024 and focused on: i) the impact of genomic medicine in PID treatment, ii) the role of digital health, including artificial intelligence, in PID care, and iii) anticipating and minimizing risks to ensure optimal access to care. Each of these topics was discussed with a long-term outlook, evaluating where we currently stand and where the field should ideally be in the next ten years. These discussions and identified recommendations are reported below.

## Part 1: Could genomic medicine revolutionize PID treatment in ten years?

Gene therapy (GT) for PID has a notable history, as severe combined immunodeficiency (SCID) was the very first disease targeted by this innovative treatment approach. In 1990, the first successful GT trial was conducted on two patients with adenosine deaminase (ADA)-deficient SCID (4), followed by X-linked SCID (SCID-X1) trials in the early 2000s (5, 6). These groundbreaking trials marked the beginning of GT’s application in human medicine as a curative treatment modality (7, 8).

Despite this pioneering work, until now, hematopoietic stem cell transplantation (HSCT) has remained the gold standard for treating severe PIDs. While HSCT has been well-validated and shown success in the treatment of leukemia and other hematological disorders alongside PIDs, early gene therapy faced important challenges such as limited efficacy and safety issues due to the risk of insertional mutagenesis, amongst other (9).

### From viral-mediated gene addition to gene editing

Gene therapy approaches primarily utilize viral vectors to deliver therapeutic genes, with gamma-retroviral vectors found to cause considerable safety risks in early clinical studies. Their insertion into the host genome could inadvertently activate oncogenes, leading to insertional mutagenesis and an increased risk of leukemia (10, 11). Enhanced viral vector engineering, such as generation of self-inactivating lentiviral vectors, and refinement of production techniques have further improved the safety profile while simultaneously increasing the efficacy and stability of gene delivery, thereby addressing the initial limitations of early GT trials (9, 12).

Following viral-mediated gene addition, more recent gene-editing technologies have significantly advanced the field of GT. Tools such as the Clustered Regularly Interspaced Short Palindromic Repeats (CRISPR)-Cas9 platform allow for precise modifications of the patient’s DNA to directly correct a genetic defect, a technology awarded the 2020 Nobel Prize in Chemistry (13). This can be achieved through insertion, deletion or modification of a gene fragment (14). While CRISPR-Cas9 based strategies typically induce double-strand DNA breaks and rely on cellular repair mechanisms, two variations of the technique were recently developed: base editing and prime editing. The former allows for one specific nucleotide base to be converted into another without the DNA backbone being affected, and the latter allows for any type of modification (insertion, deletion and substitution) to be made with potentially lower risk of off-target effects compared to classical CRISPR-Cas9 due to the single-strand break (15, 16).

Gene editing offers several advantages over viral gene addition, including a reduced risk of insertional mutagenesis, the possibility for the gene product to be endogenously regulated, resulting in physiological expression, and the potential to correct or disrupt gain-of-function mutations (9, 17, 18). Viral vector-based gene addition remains valuable, however, particularly for introducing a complete gene copy in hematopoietic stem cells (HSC), since preclinical experiments *in vivo* have demonstrated that a lower proportion of gene-edited cells is detected within the long-term HSC population (19). Moreover, several hundred treatment years of follow-up is available for patients treated with *ex vivo* HSC lentiviral gene therapy, supporting durable treatment effect and safety. The complementary use of gene addition and gene-editing techniques broadens the therapeutic possibilities, offering a valuable alternative to HSCT as a curative treatment option. For now, gene editing is just leaving the pre-clinical stages and entering the clinical trial stage, with the first trial in PID targeting X-Linked Chronic Granulomatous Disease starting in April 2024 (NCT06325709).

### T-cells in focus

Most GT applications for PID target the genomic material of HSCs. Yet, in the very first GT clinical trials, T cells were specifically isolated and manipulated (4, 20). This remains an interesting avenue for PID impacting the T cell compartment as T cells can be both more efficiently isolated and modified than HSCs, and are terminally differentiated, which reduces the risk of insertional mutagenesis (19, 21). Additionally, it requires less toxic pre-treatment conditioning compared to HSC-depleting protocols (21). Although the value of T cell gene therapy for healthcare systems remains under study, a favorable safety profile is expected to shorten hospital admissions and lessen future outpatient care. Gene editing could also benefit PID linked to T cells as it would reduce the risk of off-target mutagenesis. In addition, owing to the recent advances in chimeric antigen receptor (CAR) T cell therapy for the treatment of hematological malignancies and other diseases, the necessary infrastructure to isolate and modify T cells is increasingly available (22, 23). Nonetheless, data on long-term effects are still lacking to ascertain the long-term safety and effect durability of these therapies.

### Challenges and opportunities

Currently, the PID field has witnessed market approval of one gene therapy product, Strimvelis™, which gained market authorization by the European Medicines Agency (EMA) in 2016 as *ex vivo* HSC gene therapy for the treatment of ADA-SCID (24). Despite the great advancements and promise of GT for the treatment of PID and other rare diseases, the availability of these therapies is highly limited, mostly due to non-medical factors. A major hurdle in development of new gene treatments is the lack of commercial interest due to the significant costs associated with product development and production, combined with the small patient population, which limits the potential for financial sustainability (25, 26). While Strimvelis™ has received market authorization in Europe, in 2022 Orchard Therapeutics, the commercial producer of the therapy, discontinued production (27). The Telethon Foundation, a non-profit organization in Italy, has since then taken over the license to produce and distribute Strimvelis™. The company Bluebird bio, which specializes in gene therapy for rare diseases, decided to move away from the European market altogether due to the challenging treatment reimbursement landscape in various countries (28). This brings to light another critical hurdle, which is that current health technology assessment (HTA) and reimbursement models do not adequately capture the long-term benefits of GT as a curative treatment option when considering cost-effectiveness. Likewise, current regulatory evaluation mechanisms do not take into account the specificities of advanced therapy medicinal products (ATMP) aimed at rare diseases and are often fragmented and complex to navigate (26). As a result, investment in GT is often deprioritized, despite its clinical importance.

### Overcoming economic barriers

Possible strategies to overcome the economic barriers to GT availability include reducing production costs, implementing novel cost-effectiveness models, and stimulating GT development and manufacturing by not-for-profit organizations. While for the foreseeable future, the production cost for GT will remain considerable due to its inherently complex manufacturing process, automation and optimalisation of production processes are being explored (29). Following the trends discussed for CAR T cell production, the manufacturing of gene therapies could also be organized in regions with lower staff and facility costs (30). This approach could, in turn, help facilitate access to these products in those low-cost regions. Importantly, while GT cannot benefit from economy of scale, its personalized nature might be of benefit to the patients (26). As governments and HTA bodies are increasingly being confronted with the evaluation of ATMPs, novel approaches to assess cost-effectiveness are being explored that consider a broader set of value parameters as well as long-term benefits. The novelty of GT implies that no long-term clinical trial data or real-world evidence is yet sufficiently available to confirm effectiveness and safety of these treatments. Therefore, different outcome-based reimbursement schemes are being developed in which various factors (e.g., patient-relevant outcomes, sustained remission, etc.) continue to be evaluated post-market authorization, informing the reimbursement scheme (31, 32).

Alternatively, the field could move away from commercial production and organize both product development and manufacturing in an academic or other not-for-profit setting or move towards a model of public-private partnerships, as in the case of Strimvelis™ (33, 34). Approval and reimbursement using a hospital exemption pathway has been obtained for CAR T cell products and could be envisioned for gene therapy in the rare disease setting (34, 35).

### Overcoming regulatory hurdles

For patients to be treated with innovative therapies such as GT outside of participation in clinical trials, regulatory agencies need to review and approve their use. To do so, competent authorities and HTA committees worldwide evaluate the candidate therapies regarding their safety and effectiveness and, in the case of an already existing therapy, on their equivalency and cost (17). This model can restrict the positive evaluation of GT based on its substantially different mechanism of action from current standard treatments, disregarding the QoL improvement and event-free survival, which, while not direct therapeutic benefits, are of great importance to patients (17). Despite the potential risks associated with GT, such innovation that meets patients’ needs should be incentivized, and to accomplish this, an alignment in terms of safety and therapeutic benefit between stakeholders is essential. Patients should be involved in the design of guidelines to streamline the approval of ATMPs, which should consider their needs and QoL. Other changes to the current paradigm, like platform approvals, *i.e.*, approving a gene therapy vector once and allowing the gene of interest to be changed without incurring the approval process repeatedly, can also aid in accelerating GT development and approval (26). Collaboration between different research centers mediated by regulatory agencies could also benefit the regulatory process by promoting access to rare disease patient samples and data and reducing competition by bringing scientific and clinical efforts towards a common goal. To achieve this, referral to other regulatory agencies and sharing of expertise at a global level would facilitate the centralization of data and collaboration, allowing innovation to reach patients quicker and safely (26). Despite these opportunities for improvement, it is important to consider that market authorization does not equal availability. Thus, models to improve accessibility and sustainability for gene therapy in the long term are also needed (17, 24).

### Increasing knowledge through collaborative patient data collection

Information lies at the center of the development and approval of ATMPs such as GT. Robust data collection is essential to adequately determine GT’s impact in the short and long term and is directly dependent on stakeholders’ cooperation. Collaboration between academic institutions, not-for-profit funding agencies, policymakers, patient groups, and the pharmaceutical industry is necessary for knowledge transfer and inclusion of large cohorts with the integration of a variety of PID, population, and patient profiles, across the age spectrum (17, 24, 29). Patient-reported outcomes, beyond survival metrics, should also be included in clinical trials and in the cost-benefit analysis of GT. Since data collection and harmonization are essential to studying these therapies quicker and safely for the benefit of PID patients, IPOPI is committed to having GT and other trials listed in the PID Life Index, a tool to measure the implementation of principles of care for PID patients worldwide (36–38). This way, information regarding these studies will be centralized in an already well-known and well-used tool in the community.

While, to date, no hematological malignancies have been reported in patients with PID treated with lentiviral vectors, the risk remains (19). Patients treated with these technologies should be monitored in prospective studies to establish the potential effects of GT after a decade or more of treatment (24). Long-term follow-up of patients treated with GT, along with a close dialogue between all relevant stakeholders, is essential to ensure that GT becomes globally available and provides all possible benefits to the patients at a reasonable cost (25, 29). With this in mind, initiatives such as the one led by the Access to Gene Therapies for Rare Diseases (AGORA) Foundation are important to harmonize infra-structure, requirements and data-sharing globally in order to streamline safety assays and bring these therapies sustainably to rare disease patients, building on established global cross-border networks (26, 39). GT can revolutionize the field of PID if long-term safety and efficiency are clinically demonstrated, with an important improvement in QoL associated with a tag that healthcare systems can afford or adapt to, bearing in mind the one-time cost for a potential lifelong benefit.

See **Table 1** for the summary on the prospective (Where do we want to be? And how are we going to get there)?.

**Table 1:** Could genomic medicine revolutionize PID treatment in ten years?

Where do we want to be?	How are we going to get there?
1. Gene therapy to fulfil its potential in the field of PID. 2. Enhanced collection and access to patient samples for gene therapy research. 3. Global collaboration among countries, societies, patient groups, and registries for knowledge transfer in advanced gene therapy techniques while maintaining diversity of available products. 4. Active involvement of patients in shaping regulatory frameworks and harmonizing regulations across countries. 5. Development of economic strategies to reduce costs and promote gene therapy development and access worldwide (*e.g.* cost of vectors). 6. Integration of diverse PID diseases and populations, increasingly considering the impact of genomic therapies for adult patients. 7. Incorporate comprehensive and standardized patient-reported outcome data in clinical trials, beyond survival metrics.	1. Establish roadmaps to improve accessibility and funding sustainability for gene therapy. 2. Harmonize regulatory requirements internationally to facilitate collaboration. 3. Foster international research collaborations, stimulate interoperable or global registries (e.g., natural history studies), and utilize repositories like the IPOPI PID Life Index to centralize trial information and facilitate data accessibility. 4. Promote multi-stakeholder dialogue to develop strategies aimed at improving availability, accessibility, and affordability. 5. Viable strategies: o Explore sustainable funding models and public-private partnerships. o Collect comprehensive QoL data to demonstrate the impact of therapies o Provide support to individual academic teams to develop GT products and reach the authorization stage across multiple markets to make it accessible to more patients. 6. Conduct inclusive clinical trials with patients of all ages and establish mechanisms for long-term follow-up and data collection. 7. Mandate inclusion of patients in clinical trial design and select primary outcomes for gene therapy trials (not only restricted to PROMs/PREMs), emphasizing improvements in PID management, even in the context of partial cure.

## Part 2: The role of digital health, including artificial intelligence, in PID care 10 years from now

Medicine is witnessing the dawn of a new paradigm in which digital tools, particularly artificial intelligence (AI), can break the glass ceiling encountered in many areas (40). AI-based digital solutions have the ability to deal with large amounts of data and complex inputs while trying to augment human cognitive processes like decision-making and problem-solving. A key aspect of AI is machine learning (ML), which uses algorithms to learn from data and improve over time without being explicitly programmed for a particular function. In medicine, ML helps identify patterns in patient data, such as medical histories, imaging results, lab tests or genetic profiles, predict outcomes (i.e. treatment responders and non-responders), and improve diagnostic precision (41). A specialized subset of ML, deep learning (DL), uses multi-layered neural networks to handle complex data, such as speech (42, 43) or medical images (44), and helps with tasks like anomaly detection with high precision, such as identifying tumors in medical imaging with high accuracy. However, to maximize the potential of AI, it is essential to ensure that datasets are of high quality.

Although the implementation rate of AI-based solutions in healthcare is still low, many applications have been proposed, covering multiple areas of AI techniques. Unsupervised learning provides new insights and knowledge of diseases by finding underlying structures, patterns, and phenotypes in data without predetermined labels. Clustering algorithms, for instance, can discover subgroups within a population by grouping people with similar symptoms (45, 46). Using labelled data, supervised learning trains models for certain tasks, such as patient screening, can help research and drug repurposing and customizing medical interventions to each patient’s needs. Reinforcement learning (47) can improve individualized treatments (48) by learning effective strategies through reward-based feedback. Natural language processing (NLP), a branch of AI that focuses on processing and producing human language, may also lessen the workload of healthcare professionals (HCP) by automating the collecting and processing of clinical notes and patient data and enabling the human interaction aspect during medical consultations (49). NLP can also extract phenotypes from raw medical data (50–52), as well as enable early risk assessment of a variety of diseases (53).

### Shifting the paradigms of PID: from delayed diagnostic odyssey to personalized management

AI technology is revolutionizing medicine by enabling advanced data analysis, personalizing treatments, and optimizing healthcare delivery, allowing digital systems to process vast datasets efficiently, generate actionable insights, and address complex medical challenges. All these features can be particularly helpful in the setting of rare diseases like PIDs, where patients often experience a diagnostic odyssey before finding an adequate diagnosis, treatment and management by specialized HCP (54–56). AI’s capacity to examine large datasets and spot minute patterns can become an important asset to aid towards earlier diagnosis (57–59), reducing misdiagnosis (60), and improving treatment (61). By incorporating genomic data, AI could lead to earlier and more accurate identification of at-risk individuals and tailor treatment plans, leading to more effective and efficient and truly personalized medical care. Finally, AI can be used to speed up drug development, discovery, or even drug repurposing, especially of interest in rare diseases like PID (62).

Therefore, AI can potentially shift the current paradigm of healthcare delivery. However, for this future to become the present, many challenges must still be addressed. AI feeds from data; hence, the success of AI-based solutions relies on the availability of large amounts of high-quality data that are stored and recorded in a usable and accessible way (63). Ensuring data privacy, integrating AI with existing healthcare systems, and making AI tools accessible and user-friendly for both patients and healthcare providers, all while navigating a changing regulatory framework, are critical steps toward realizing the full potential of AI in medicine.

### Interoperability and the globalization of healthcare solutions

Envisioning the role of digital health and AI in PID care a decade from now brings to light several challenges and potential solutions highlighted as key points in **Table 2**. Most of these challenges are associated with the opportunistic and unplanned digitalization in which the implementation of electronic health records (EHR) has grown. This uncoordinated development has led to a fragmented landscape with disparate data sources that are sometimes misaligned and unable to form a comprehensive view of a patient (64, 65). This disarray hinders the effective use of AI solutions in healthcare, as seamless data integration is crucial for the accurate analysis and utilization of health information (66).

**Table 2:** The role of digital health, including AI in PID care 10 years from now.

Where do we want to be?	How are we going to get there?
1. EHRs composed of high-quality, accessible and harmonized data. 2. Improved data collection methods. 3. Effective use of AI in healthcare (“‘real-word data”) to empower patients and HCP in disease diagnosis, treatment, and management. 4. Ensured a central role for data privacy, ethics and informed consent. 5. Reduced underdiagnosis and diagnostic delay while improving the accuracy of diagnosis and prognosis. 6. Scaled and democratized AI in healthcare.	1. Establish standardized data formats and protocols to ensure consistent data quality and accessibility. Involve scientific societies and patient advocacy organizations (PAOs) to ensure the unity in choosing a common standardized language (e.g. Observational Medical Outcomes Partnership). 2. Complement free text and structured data with other reliable data collection methods, such as contact and contactless devices and audio recordings. 3. Multiple venues possible: • Leverage AI to improve the quality of medical records and support continuous quality improvement, thus accelerating learning within health systems. • Make data more accessible by enhancing transparency and giving control to patients while providing accessible and reliable tools to HCP. • Use AI powered/automated tools to improve monitoring, registry and analysis of pediatric and adult individuals’ metrics as well as PROMs. • Use AI tools to increase patient knowledge and involvement, and the humanization of healthcare by setting up patient centered medical goals. • Gather and compile data on current trials and populate tools such as PID Life Index (see **Table 1**) for both patients and healthcare providers and registries to recruit patients in clinical trials. 4. Implement federated learning and advanced AI techniques to protect patient data and enhance privacy while developing clear and consistent consent processes tailored to different legal frameworks, ensuring patients are well-informed about data usage. 5. Use already existing data both in primary care and hospital settings with constant learning models to identify underdiagnosed or misdiagnosed individuals, reduce diagnosis delay and irreversible organ damage, and provide better insight on prognosis for patients and families. 6. Utilize AI to democratize healthcare by providing globally accessible diagnostic tools and insights, especially in low-income regions, and enhance data capture and sharing between patients and HCPs globally.

Central to solving these issues are clinical information models and the concepts of semantic and syntactic interoperability. Semantic interoperability refers to the ability of different healthcare systems to exchange data with unambiguous, shared meaning, ensuring that the information is understood consistently across different systems. Syntactic interoperability, on the other hand, focuses on the format and structure of data exchange, enabling different systems to interpret the data correctly. Achieving both types of interoperability is key to creating a cohesive and functional global healthcare data ecosystem.

Furthermore, standardizing data formats and protocols is essential to guarantee consistent data quality and accessibility. Involving patient advocate organizations and scientific societies in the selection of a common data model, like the Observational Medical Outcomes Partnership (OMOP (67)), could help promote more efficient data integration and medical research. Additionally, AI-powered and automated tools can enhance the monitoring of both pediatric and adult health metrics, as well as Patient-Reported Outcome Measures (PROMs), offering a more comprehensive approach to patient care. The role of PROMs, along with strategies for their broader implementation and standardization in clinical care and research, was a key focus of discussion at the 2023 Global Multi-Stakeholders’ Summit (2).

Even while interoperability solutions can reduce fragmentation to some extent, data will not be used extensively and wisely until data storage quality improves (68), ensuring it is precise, comprehensive, and formatted. Even advanced AI algorithms are unable to provide trustworthy and relevant insights in the absence of high-quality data (69). The potential advantages of AI in healthcare are undermined by poor data quality, which limits AI reliability. Data quality and true interoperability could significantly improve by creating standardized data formats and incorporating important stakeholders in the adoption of agreed and unified standards. By doing this, a strong basis for AI applications will be established, allowing for the more efficient and thorough utilization of healthcare data to enhance patient outcomes and progress medical research.

### More information to raise the standard of PID care

Data accessibility and dependability difficulties are expected to intensify as healthcare data volume and variety rise. To fully harness the power of data for better patient treatment and outcomes, more phenotypic and genetic data needs to be gathered, and NLP should be used to facilitate conversion of unstructured data from patient records, clinical notes, and other text sources into structured formats that can be integrated by AI systems. Personalized therapy is possible when based on, among other factors, well-curated genetic data analyzed by powerful algorithms, which offer insightful information about each patient’s unique profile in the context of enhancing their treatment and QoL.

Using audio recordings and NLP approaches, AI can also improve how patient-provided information is collected, stored and used during consultations. These tools would be able to automatically record and transcribe conversations, recognize important information, and arrange, combine, and standardize this data in EHR. By improving data consistency, lowering human error linked with manual documentation, and enhancing interoperability, professionals will have more time to concentrate on patient care and increase humanization (66). This could significantly improve the overall standard of care while simultaneously increasing the accessibility and accuracy of patient data.

Another potential benefit of AI regarding the uptake of information from patients is the integration and use of data from contact and contactless devices, such as wearable devices (i.e. smartwatches and fitness trackers, amongst others) (70). These devices, which are part of the broader network known as the Internet of Things (IoT), are growing rapidly in type and number, generating potentially valuable clinical information. Unlike traditional statistical methods, AI is capable of ingesting complex sensor data to make predictions. However, sophisticated systems for data protection, management and storage are required due to the increasing variety and volume of data, as well as rigorous privacy data protection regulations. Additionally, the uncontrolled growth of wearable devices and sensors poses a risk of data fragmentation because of the lack of harmonization in the clinical information models behind each device. Therefore, for these devices to be incorporated, harmonization would be needed.

### Privacy concerns

Aside from technological challenges, the increasing role of data in healthcare poses ethical and regulatory challenges. Privacy concerns are paramount when it comes to health data collection (71). Health data are highly sensitive, and their misuse can lead to serious consequences for individuals. The regulatory landscape of health data protection is rapidly evolving to address these concerns, with new laws and guidelines aimed at protecting patient privacy while allowing for the beneficial use of data. Strict compliance with these regulations requires ongoing vigilance and adaptation by healthcare organizations and technology providers.

### Tackling the challenges

Taking up the aforementioned challenges, several efforts have emerged in response to the data fragmentation that currently limits the potential of AI in healthcare. Two remarkable examples of these efforts are the PIDCAP (72) and the Dr. Warehouse (73) projects, both aimed at integrating routine data and leveraging their potential in day-to-day practice. The PIDICAP project aims to enhance coordination between primary care and referral facilities in Catalonia to enable earlier PID diagnosis. Similar to SPIRIT^®^ Analyzer and other initiatives (58, 74), using a computerized diagnostic algorithm, the EHR of primary care are screened for PID warning signs to automatically identify individuals at high risk of PID and refer them to the nearest reference center, ultimately shortening the diagnostic time and improving disease outcomes. Similarly, the Dr. Warehouse project aims to achieve an effective data integration of narrative reports in clinical settings by transforming unstructured clinical narratives into structured data that AI systems can access (51). This approach allows the use of additional and valuable healthcare information for both research and routine care, which would otherwise go unnoticed. Furthermore, the uptake of data from textual reports allows for a better contextualization of health data, thus increasing their quality.

Finally, to assure equity, AI-based solutions should be accessible to all countries and all population groups based on their needs. This implies not only a broad distribution of this technology but also building the structural conditions for it to work in low- and middle-income countries. This includes investing in healthcare infrastructure, training HCP, and creating policies that support the adoption of AI in healthcare worldwide. Without these efforts, the benefits of AI in healthcare will remain concentrated in wealthier nations, exacerbating global health disparities.

While AI holds great promise for transforming healthcare, realizing its full potential requires overcoming significant challenges in data collection, integration, quality, accessibility, and privacy. In the meantime, while all these advancements are being developed, data being collected should be used effectively to progressively move towards a model of a learning healthcare system. This approach involves continuously analyzing existing data to generate insights that can improve PID patient care in real-time. By leveraging traditional statistical models and ML techniques on available data, HCP can identify patterns, predict outcomes, and personalize PID treatments even within the constraints of imperfect data systems. This iterative process not only enhances current patient care but also creates a feedback loop where each clinical encounter contributes to the overall knowledge base, steadily improving the healthcare system’s effectiveness and efficiency. This gradual shift towards a learning healthcare system ensures meaningful progress while addressing the broader challenges of data management and integration.

See **Table 2** for the summary on the prospective (Where do we want to be? And how are we going to get there)?.

## Part 3: Anticipating and minimizing future risks to ensure optimal access to care

The history of healthcare shows a need for constant surveillance and improvement of pre-established systems and standards. For example, nowadays, the development of plasma-derived medicinal products (PDMP) involves multiple viral inactivation steps due to incidents in the 1980s and 1990s that led to patients using such treatments contracting HIV and hepatitis. The COVID-19 pandemic is an example of a recent event with a broad impact that revealed vulnerabilities in the plasma supply chain, affecting patients globally. PID patients are vulnerable to events that affect public health and are dependent on life-long treatments such as PDMPs, including immunoglobulin replacement therapy (IgRT). Therefore, threats that present risks to the availability of and access to their treatments and well-being should be identified and minimized to avoid increasing morbidity and mortality in this group.

### Counterfeit medicine and suboptimal therapies, affordability and availability

Challenges exist at multiple levels in the care of PID patients and taken together against a backdrop of supply issues and rising costs of medicine, these may grow out of proportion if not tackled in a timely and adequate manner. In recent years, patient organizations and other stakeholders have reported a worrying increase in the circulation of counterfeit medicines, particularly in LMICs. The development of alternative small-scale production of PDMPs, including immunoglobulin therapies, has also raised concerns as to their safety and efficacy (75, 76). Counterfeit medicines are deliberately mislabeled in regards to their composition or source, deceiving both patients and HCP into using a substandard treatment that may lead to treatment failure and risk of death (77). Regulatory authorities must ensure that only rigorously evaluated, quality treatments reach patients, as counterfeited drugs are a recognized issue affecting multiple countries globally (77). A constant evolution of quality standards and controls while maintaining treatment affordability is crucial, ensuring a balance without compromising the safety and accessibility of these essential medicines. Measures to improve manufacturing capabilities and regulatory oversight, aiming for global sufficiency in PDMPs based on regionally balanced plasma collection, would also significantly improve access and, as such, tackle the issue of suboptimal products and counterfeit medicines. Investment in regulatory systems and reimbursement policies is crucial to minimize this issue.

### History to support innovation

Clinical trials are necessary to verify the safety and efficacy of innovative treatments, but traditionally, limited additional information is gathered. Frequently, data on QoL or other outcomes are perceived as secondary when in fact they provide additional data on treatment effectiveness and real-life impact on patients. Similarly, approved drugs that are used off-label usually do not have studies to report short or long-term effects on a population of rare conditions like PID, which often are not featured as a cohort in clinical trials. The rarity of PID conditions, of which there are now 555, according to the 2024 classification by the International Union of Immunological Societies Expert Committee, leads to fewer relevant clinical studies for each individual disease (78). The inclusion of PID patients in clinical trials of drugs and medical devices with other indications is important while the follow-up and pharmacovigilance of medicines already used by these patients are crucial to provide adequate care and have information regarding long-term use. Collecting more information during clinical trials and routine treatment of PID patients is sure to aid in establishing registries and models to evaluate current guidelines and treatments, as well as improve outcomes. A good example is the recent studies demonstrating HSCT is a sustainable and cost-efficient treatment for XLA (79). As HSCT is increasingly being demonstrated as a viable curative option, GT and other innovative therapies may also become viable, cost-effective therapeutic options for some specific types of PIDs and associated conditions. Still, as long as these treatments are not readily available, omics and other techniques can support patient stratification and appropriate use of immunoglobulins and other prophylactic medicines, optimizing their effectiveness and use.

### Stakeholders’ roles in optimizing care

Equitable access to a range of therapies and personalized care is, therefore, crucial to ensure optimal care for patients living with a PID. For this to happen, an accurate diagnosis as early as possible is key so that the best therapeutic options can be promptly selected. This model of care is possible if all stakeholders align their goals and cooperate. Studies that collect and centralize long-term outcome data on the natural history of PID patients with the addition of other routinely monitored markers can help stakeholders in multiple instances. Regulators would be able to adjust their policies and recommendations whilst reimbursement schemes could be adequately adapted (80). Improved regulatory frameworks and HTA systems that aptly evaluate specialty medicine, which is often heterogeneous and groundbreaking, are crucial for the optimization of care (80). In parallel, incentives to invest in innovation and essential medicines are imperative to avoid situations in which new ATMPs are withdrawn from the market after approval while basic therapies become less available due to the lack of economic viability (*e.g.* antibiotics). Researchers and the industry could benefit from clear regulatory streamlined evaluation models, which would facilitate the evolution of the pharmaceutical business model from high-volume, low-margin drugs to more personalized treatments (80). Policymakers and patient organizations play pivotal roles in articulating the safety requirements and economic factors with the real-life evidence and necessities from the target populations of these treatments. Collaboration and standardization in data collection, treatment guidelines and regulatory standards would help create policies that improve access to elevated standards of care including personalized medicine that reduces the costs associated with disease management and therapy inconsistencies by ensuring appropriate use of resources. This collaboration should be based on public-private partnerships to ensure effective and equitable advantages to all participants. Lastly, a significant investment in education across all stakeholders would also contribute to the speed of this development, representing an initial investment to provide a long-term, affordable, burden-free QoL.

See **Table 3** for the summary on the prospective (Where do we want to be? And how are we going to get there)?.

**Table 3:** Anticipating and minimizing future risks to ensure optimal access to care.

Where do we want to be?	How are we going to get there?
1. Improved global patient access to safe, effective, and continuous Ig therapy. 2. Better long-term outcome data on the natural history of PID patients (including adults), and comprehensive indicators (e.g., biomarkers, omics data, health economic outcome measures) that allow for the development of predictive tools and patient databases, potentially through AI-powered solutions, to refer the correct patients to curative therapies such as HSCT, at the right time. 3. Enhanced effectiveness of policies aimed at balancing affordability, access, and efficiency of healthcare to improve health equity. 4. Improved regulatory framework and health technology assessment system, apt for specialty medicine evaluation. 5. Ensured adequate ecosystem for reliable availability of targeted therapies to PID patients. 6. Ecological footprint considered in effective patient-centered legislation without compromising patient access to care.	1. Harmonization of best practice recommendations and safety guidelines for PDMPs, empowering sustainable plasma collection models while considering region-specific needs. 2. Establish more PID reference centers, including adult PID clinics, supported by a network of specialized centers, international exchange programs, and telemedicine, to leverage real-world data, and registry data to determine the most suitable treatment approach for different patient groups. 3. Modify assessment framework to capture broader societal values (e.g., long-term productivity and absence of medical need, family spillovers, rarity and severity of disease, scientific spillover from innovative technology) within a dynamic opportunity-based model. This model should consider wider health promotion and preventive measures. 4. Stimulate patient-driven and patient-centric collaborative international studies for better decision-making, encompassing estimates on QoL improvements and health-economic factors. 5. Streamline the licensing process for medicines used off-label for a limited set of indications, including (ultra-)rare diseases, and integrate these uses into PID registry goals. 6. Promote patient-centered approach in the assessment and decision-making relating to upcoming ecological policies to ensure the most balanced approach is met to safeguard the environment whilst protecting patient access to treatment.

## Conclusions and recommendations

In light of the rarity, diversity and costs associated with PID, there is a need for a comprehensive approach to care and access to treatment, facilitating curative options like HSCT and GT based on real-world evidence with the prospect of leading to personalized medicine based on patients’ needs. In addition, there is a need to ensure more equitable access to safe and effective Ig therapies. To accomplish this, efforts must prioritize collaboration between academia, industry, regulatory authorities, and patients to achieve sustainable progress through different aspects:

- Public-private partnerships that involve all stakeholders are beneficial to the swift development and monitoring of new technologies.
- Innovation should be regarded as an investment to provide patients with an improved QoL, focusing on multiple outcomes instead of efficacy or safety alone.
- Inclusion of patient data and centralization of information, including natural history studies and clinical research targeting adult patients, through platforms such as the PID Life Index to facilitate data accessibility and dissemination, aiding the development of an AI tool to integrate and use this information.
- Use a common data model (e.g. OMOP) to allow data harmonization globally, ensuring that PID research and its benefits are equitably distributed across countries with varying income levels.
- Practical guidance and cooperation in developing and approving advanced therapies, including outcome-based or other strategies to establish economically viable curative plans.
- Strengthening regulatory oversight to ensure access to safe and effective PDMPs such as IgRT therapies.
- Multinational collaborations would strengthen access to medicines, and data sharing would lead to improved HTA.
- The role of AI and the opportunities it presents to facilitate and improve current hurdles related to PID diagnosis and management, as well as humanization and patient empowerment.

While these collaborations are implemented, patients should remain at the center of relevant decision-making discussions with the goal of ensuring equitable access to the best care available. Patient advocacy organizations play a central role in driving policy changes and awareness of these important causes at a global level. The IPOPI Global Summit’s stakeholders remain committed to working together towards the common goal of assuring that current treatments are accessible, safe, and affordable for PID patients all over the world while implementing innovative personalized technologies. The recommendations arising from this meeting are intended for all relevant stakeholders in the field of PIDs and associated conditions, including, importantly, the regional PID scientific societies.

## Data Availability

The original contributions presented in the study are included in the article/supplementary material. Further inquiries can be directed to the corresponding author/s.

## In Memoriam

We would like to dedicate this manuscript to the memory of our co-author Jose Drabwell, who sadly passed away before it was published.
